# Supplementary material for: The causal role of circulating inflammatory markers in osteoporosis: a bidirectional Mendelian randomized study
Source: Front Immunol. 2024 Jul 18;15:1412298. doi: 10.3389/fimmu.2024.1412298 (PMC11291241; doi:10.3389/fimmu.2024.1412298)
Supplement: Supplementary file 1 [file DataSheet_1.zip › supplementary material/CIM-OSTEOPOROSIS /GCST90274816.csv_forest.pdf]

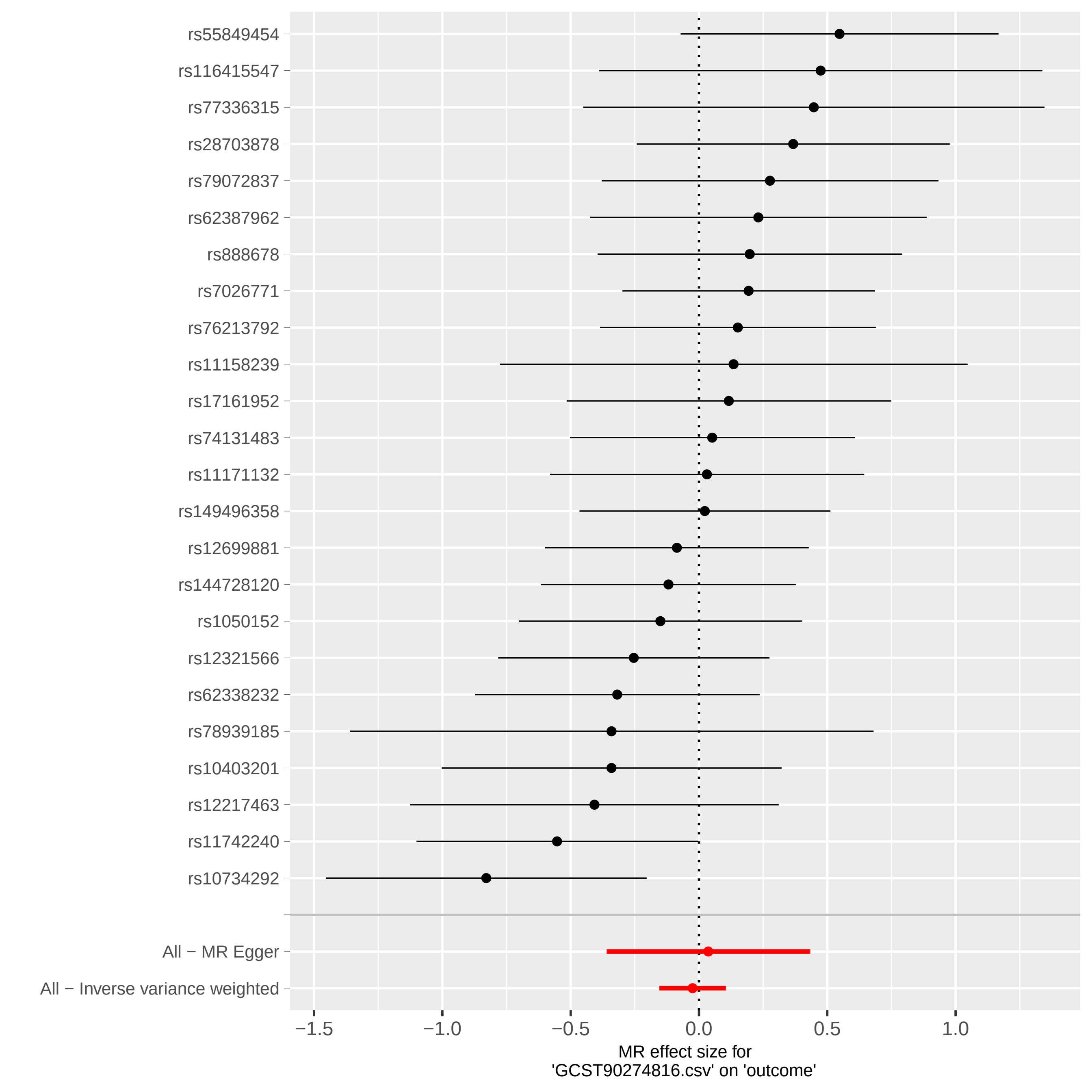
rs55849454
rs116415547
rs77336315
rs28703878
rs79072837
rs62387962
rs888678
rs7026771
rs76213792
rs11158239
rs17161952
rs74131483
rs11171132
rs149496358
rs12699881
rs144728120
rs1050152
rs12321566
rs62338232
rs78939185
rs10403201
rs12217463
rs11742240
rs10734292
All − MR Egger
All − Inverse variance weighted
−1.5
−1.0
−0.5
0.0
0.5
1.0
MR effect size for
'GCST90274816.csv' on 'outcome'
